# Evolution and Antimicrobial Resistance Profiles of *Klebsiella* spp. Infections in Companion Animals in the Iberian Peninsula

**DOI:** 10.3390/antibiotics15070678

**Published:** 2026-07-10

**Authors:** María Jiménez-Serrano, Anna Vidal, Inma Duran, Chiara Seminati, Laila Darwich

**Affiliations:** 1 Department of Sanitat i Anatomia Animals, Veterinary Faculty, Universitat Autonoma de Barcelona, 08193 Cerdanyola del Vallès, Spain; 2 Departamento de Veterinaria de Laboratorios Echevarne, 08037 Barcelona, Spain

**Keywords:** *Klebsiella pneumoniae*, *Klebsiella oxytoca*, dogs, cats, AMR evolution, Spain

## Abstract

Background/Objectives: Antimicrobial resistance (AMR) in companion animals is an increasing concern within the One Health framework, particularly regarding opportunistic pathogens such as *Klebsiella* spp. This retrospective study evaluated the epidemiology, antimicrobial susceptibility profiles, and temporal resistance trends of *Klebsiella* spp. infections in dogs and cats across the Iberian Peninsula. Methods: A total of 809 clinical isolates collected between 2016 and 2024 and submitted to a private diagnostic laboratory in Barcelona were analysed. Results: *Klebsiella pneumoniae* was the predominant species (70%), more frequently identified in cats (76%) than in dogs (68%). Dermatological and respiratory samples exhibited the highest prevalence of multidrug-resistant (MDR) isolates. Overall MDR prevalence was high, particularly in cats (51.1%; 95% CI 41.1–60.9%) compared with dogs (38.4%; 95% CI 34.1–42.8%) although it was not statistically significant. *K. pneumoniae* generally exhibited higher resistance rates than *K. oxytoca*, particularly to amoxicillin/clavulanic acid, first-/second-generation cephalosporins, third-/fourth-generation cephalosporins (3/4th GC), fluoroquinolones, and tetracyclines. In both bacterial species, resistance rates were consistently higher among feline isolates. In contrast, aminoglycosides and phenicols retained high activity against most isolates. Temporal analysis revealed a significant increasing resistance trend to amoxicillin/clavulanic acid, which is particularly concerning given the widespread use of this antimicrobial as a first-line treatment in small animal practice. However, resistance trend to aminoglycosides showed a significant decline. No significant temporal changes were detected for 3/4th GC and fluoroquinolones, suggesting the persistence of resistant populations within companion animals. Resistance to aminoglycosides and phenicols remained comparatively low in this study. Whereas critically important category B antimicrobials, such as 3/4th GC and fluoroquinolones, exhibited low to moderate effectiveness, raising concerns about their empirical use. Conclusions: These findings highlight the substantial AMR and MDR burden of *K. pneumoniae* in companion animals in the Iberian Peninsula and reinforce the need for prudent antimicrobial use, routine susceptibility testing, and integrated One Health surveillance strategies.

## 1. Introduction

Antimicrobial resistance (AMR) is recognized as one of the most significant threats to global public health. In 2021, bacterial AMR was directly responsible for an estimated 1.14 million deaths worldwide and associated with an additional 4.71 million deaths, highlighting its substantial impact on global health systems [1]. The increased use of antibiotics during the COVID-19 pandemic, including critically important agents, may have further contributed to the selection and dissemination of resistant bacteria [2,3]. Thus, the emergence and dissemination of multidrug-resistant bacteria (MDR) compromise treatment efficacy, increase healthcare costs, and threaten the sustainability of modern medicine. Consequently, AMR has become a major priority within the One Health framework, which recognizes the interconnectedness of human, animal, and environmental health.

Although surveillance efforts have traditionally focused on human medicine and food-producing animals, because of their role in transmitting resistant bacteria through the food chain [4], increasing attention is being directed toward companion animals as potential reservoirs and disseminators of AMR bacteria [5]. Dogs and cats live in close contact with humans, creating opportunities for the bidirectional exchange of bacterial pathogens and resistance determinants. Recent evidence from Europe indicates that companion animals may play an important role in the maintenance and spread of clinically relevant resistant bacteria, emphasizing the need to include veterinary settings in integrated AMR surveillance programs [6,7,8]. Furthermore, antimicrobial use in companion animal practice continues to exert selective pressure that may contribute to the emergence and persistence of resistant bacterial populations. A recent review highlighted the growing importance of monitoring antimicrobial resistance in companion animals and underscored the need for harmonized surveillance strategies across European countries [9].

Among the bacterial species of concern, *Klebsiella* spp. have emerged as important opportunistic pathogens in both human and veterinary medicine [10,11]. Members of this genus are ubiquitous in the environment and commonly colonize the mucosal surfaces of mammals including humans, horses, and swine, which commonly colonize [12,13]. Furthermore, *Klebsiella pneumoniae* belongs to the ESKAPE group of pathogens, which are characterized by their remarkable ability to accumulate antimicrobial resistance and cause difficult-to-treat infections. The detection of multidrug-resistant, extended-spectrum β-lactamases (ESBL)-producing, and occasionally carbapenem-resistant *K. pneumoniae* lineages in companion animals, including clones closely related to those circulating in human healthcare settings, reinforces the value of dogs and cats as potential reservoirs and sentinels of emerging AMR within a One Health context [14]. Moreover, genetically indistinguishable resistant strains have been identified in dogs, cats, and their owners sharing the same household, supporting the occurrence of interspecies transmission [14,15]. Consistently, *K. pneumoniae* isolates belonging to the human epidemic clone ST11 and exhibiting high-level aminoglycoside resistance have also been recovered from companion animals in Spain [16].

The growing interest in *Klebsiella* spp. in companion animals is also supported by their increasing clinical relevance in veterinary medicine. Although *Escherichia coli* remains the most frequently isolated Enterobacterales species in dogs and cats, *Klebsiella* spp. are regularly associated with urinary tract infections, wound infections, otitis, respiratory disease, and other opportunistic infections in small-animal practice [14,17,18,19]. Studies from the Iberian Peninsula have identified *Klebsiella* spp. among the bacterial pathogens recovered from clinical samples of dogs and cats and have documented concerning levels of resistance to critically important antimicrobials, particularly among feline isolates [20]. These findings highlight the potential role of companion animals as reservoirs of clinically relevant resistant clones and reinforce the importance of including this species in One Health surveillance frameworks.

Despite increasing recognition of the epidemiological importance of *Klebsiella* spp. in companion animals, information regarding the long-term evolution of antimicrobial resistance patterns in veterinary isolates remains limited. Most available studies have been restricted to specific geographic regions, short study periods, or specific resistance phenotypes, hindering a comprehensive assessment of temporal trends. Therefore, the present study aimed to investigate the distribution, antimicrobial susceptibility patterns, and temporal resistance trends of *Klebsiella* isolates recovered from dogs and cats in Spain, Portugal, and Andorra over a period from 2016 to 2024. By providing updated regional data, this study seeks to improve understanding of AMR dissemination in companion animals and support the development of integrated One Health surveillance and antimicrobial stewardship strategies.

## 2. Results

A total of 809 *Klebsiella* spp. isolates were recovered during the study period, including 670 from dogs and 139 from cats. *Klebsiella pneumoniae* was the predominant species, accounting for nearly 70% of all isolates (563/809), and was more frequently identified in cats (76%) than in dogs (68%). *Klebsiella oxytoca* was the second most prevalent species in both animal species, representing approximately one-fifth of the isolates (20% in dogs and 18% in cats). In contrast, *K. aerogenes* and *K. variicola* were detected at substantially lower frequencies, together accounting for less than 10% of isolates in either host species. The distribution of *Klebsiella* species was broadly similar between dogs and cats, although *K. pneumoniae* showed a greater predominance among feline isolates (Table 1).

**Figure 1:**
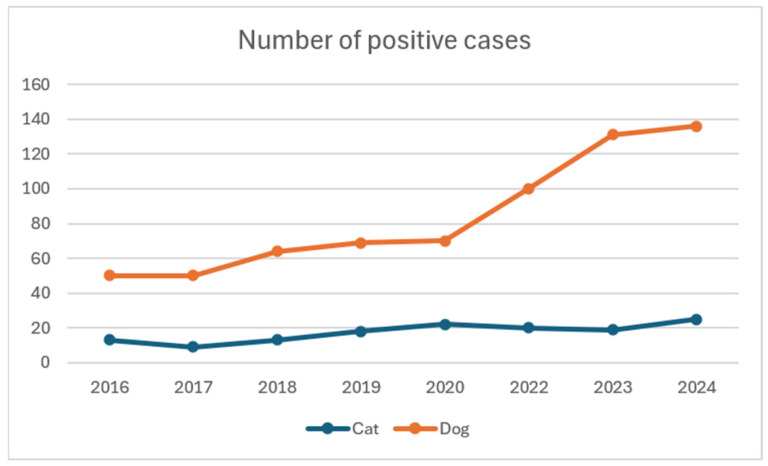
Number of *Klebsiella*-positive isolations along the study period.

The number of *Klebsiella*-positive isolations increased over the study period in both dogs and cats (Figure 1). Although the trend was substantially more pronounced in dogs showing a numerically greater increase, the difference in trend between species was not statistically significant (*p* = 0.161). In dogs, the annual number of positive isolations remained relatively stable at approximately 50 cases in 2016–2017, followed by a gradual increase to around 70 cases in 2019–2020. A marked rise was subsequently observed, reaching 100 cases in 2022 and increasing further to 131 and 136 cases in 2023 and 2024, respectively. In cats, the number of positive isolations was consistently lower throughout the study period. Annual counts fluctuated between 9 and 25 cases, decreasing from 13 cases in 2016 to 9 cases in 2017, before increasing to 22 cases in 2020. Thereafter, a slight decline was observed in 2022–2023 (keeping close to 20 cases), followed by an increase to 25 cases in 2024, the highest value recorded for cats (Figure 1).

**Figure 2:**
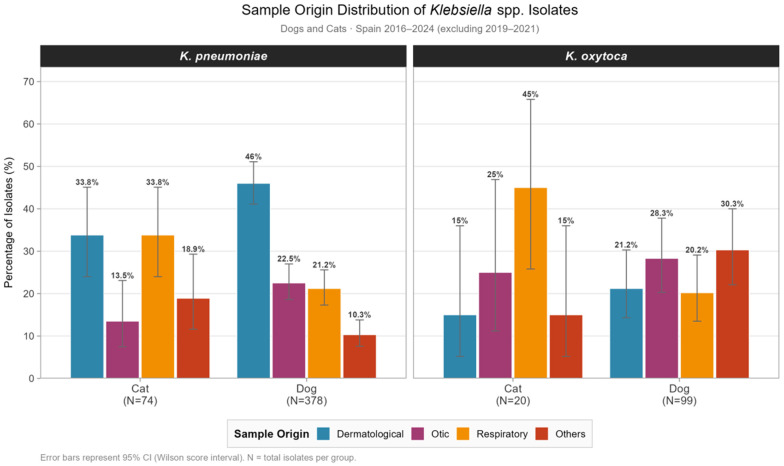
Distribution of *Klebsiella* isolates grouped by sample origin in dogs and cats. “Others” category comprises minor cases such as urinary, gastrointestinal, reproductive, bone, joint, cerebrospinal fluid, and ocular infections.

The distribution of sample origins differed significantly between dogs and cats (χ^2^(df = 3) = 12.989, *p* = 0.0047). Dermatological and otic samples were more frequent in dogs (40.9% and 23.7%, respectively), whereas respiratory samples were proportionally more common in cats (36.2% vs. 21.0% in dogs), reflecting known differences in the clinical presentation of *Klebsiella* infections between species. The distribution of sample origin of *K. pneumoniae* and *K. oxytoca* isolates in dogs and cats is presented in Figure 2.

**Figure 3:**
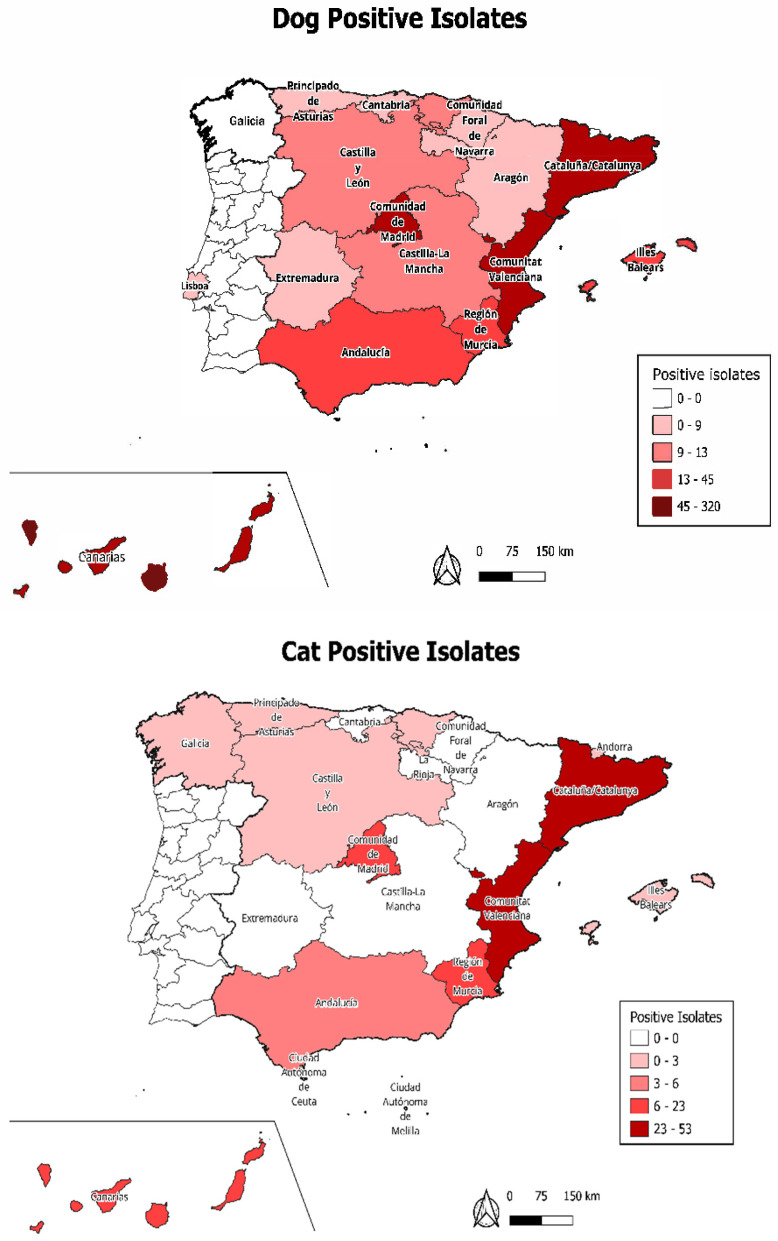
Geographical distribution of canine and feline positive cases studied in The Iberian Peninsula. Spain: Andalucía, Andalusia; Aragón, Aragon; Principado de Asturias, Asturias; Illes Balears, Balearic Islands; Canarias, Canary Islands; Cantabria, Cantabria; Castilla la Mancha, Castile–La Mancha; Castilla León, Castile and Leon; Cataluña/Catalunya, Catalonia; Comunitat Valenciana, Valencian Community; Extremadura, Extremadura; Galicia, Galicia; Comunidad de Madrid, Community of Madrid; Región de Murcia, Region of Murcia; Comunidad Foral de Navarra, Navarre; La Rioja, La Rioja. Principado de Andorra: Andorra. Portugal: Lisboa, Lisbon.

The data revealed a notable concentration of positive cases in the eastern and southern regions of Spain, particularly in Catalonia, Valencian Community, Region of Murcia and Andalusia, across all animal species (Figure 3). Additionally, Community of Madrid in central Spain presented also a high frequency of cases. Other regions such as Castile and León and Castile–La Mancha, reported moderate levels of positive isolates, while northern and western territories, including Galicia, Asturias, and Extremadura, showed lower case numbers. Canarias also exhibited a considerable number of positive isolates (Figure 3).

**Figure 4:**
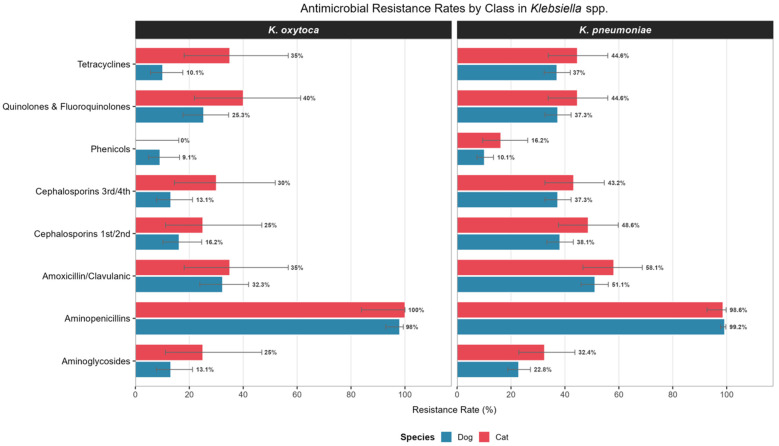
Frequencies of antimicrobial resistance by class and animal group.

Overall, *K. pneumoniae* generally exhibited higher resistance rates than *K. oxytoca* across most antimicrobial classes, particularly to amoxicillin/clavulanic acid, first-/second-generation cephalosporins, third-/fourth-generation cephalosporins, quinolones/fluoroquinolones, and tetracyclines (Figure 4). In both bacterial species, resistance rates were consistently higher among feline isolates than canine isolates. The greatest differences between cats and dogs were observed for tetracyclines and cephalosporins, whereas resistance to aminopenicillins was uniformly high regardless of host species. Phenicol resistance remained comparatively low in both *Klebsiella* species (Figure 4).

Antimicrobial susceptibility testing indicated that in cats as in dogs, polymyxins and aminoglycosides represent the most effective antibiotic groups across sample types (Table 2). Although 95–100% of the isolates from dogs were sensitive to polymyxins, it should be emphasized that these agents belong to category B and must be preserved as last-resort drugs for human medicine. Thus, considering the high levels of susceptibility in category C drugs such as aminoglycosides and phenicols, these agents should be recommended as first option for treating *K. pneumoniae* infections in dogs and cats.

Of major concern, category B antimicrobials such as third- and fourth-generation cephalosporins and fluoroquinolones generally showed low sensitivity rates (45–60%) in both dogs and cats, with the exception of otic infections in dogs, where sensitivity reached 70%. In addition, the efficacy of polymyxins in cats was lower than in dogs, particularly in dermatological and respiratory samples (Table 2). Furthermore, the intrinsic resistance of *K. pneumoniae* isolates to aminopenicillins, makes this antimicrobial class ineffective for treating these infections in dogs and cats. The addition of Clavulanic Acid only slightly improved susceptibility, but effectiveness consistently remained at or below 50% in both species (Table 2).

Among *Klebsiella* isolates, MDR prevalence was higher in cats (51.1%; 95% CI 41.1–60.9%) than in dogs (38.4%; 95% CI 34.1–42.8%). Although this difference was statistically significant before correction (Fisher’s exact test, *p* = 0.029), it did not reach significance after Bonferroni adjustment for multiple comparisons and should therefore be interpreted with caution (Table 3).

Temporal trends in antibiotic resistance were assessed from 2016 to 2024, except for the years 2019 to 2021 that were not recorded due to informatic issues in the lab. Logistic regression analysis revealed that most antimicrobial classes did not show a statistically significant temporal trend after Bonferroni correction (Table 4), with the exception of two antibiotic classes: aminoglycosides and amoxicillin/clavulanic acid.

**Figure 5:**
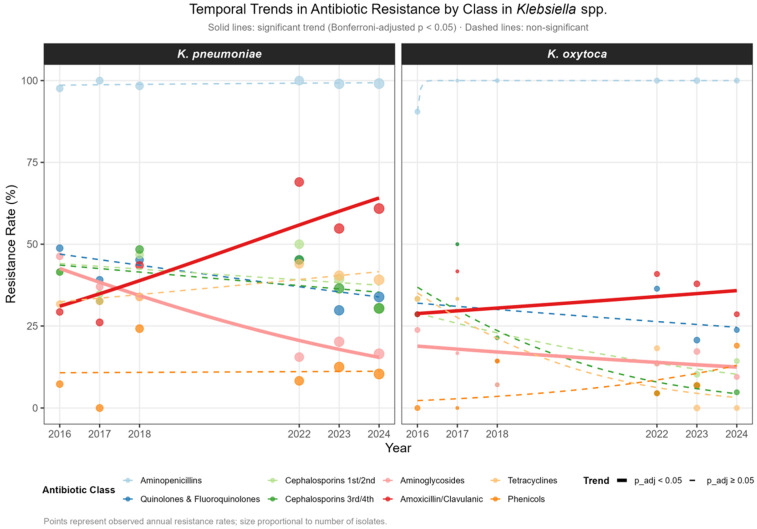
Temporal trends in antibiotic resistance were assessed using logistic regression models with calendar year as the continuous independent variable and resistance status (binary outcome, R only per EUCAST criteria) as the dependent variable for each antibiotic class. Odds ratios (ORs) per year with 95% confidence intervals were estimated using the logit link function. *p*-values were adjusted for multiple comparisons using the Bonferroni method (n = 11 comparisons). Analyses were performed for all *Klebsiella* spp. isolates combined and stratified by bacterial species for descriptive purposes. Figures display solid regression lines for statistically significant trends (Bonferroni-adjusted *p* < 0.05) and dashed lines for non-significant trends.

Aminoglycosides showed a significant decreasing trend in resistance over the study period (OR = 0.863 per year, 95% CI 0.809–0.921, *p* < 0.001), indicating an annual reduction of approximately 14% in the odds of resistance. This trend was consistent across both *K. pneumoniae* and *K. oxytoca* as observed in Figure 5. On the other hand, Amoxicillin/Clavulanic acid showed the opposite pattern, with a significant increasing trend (OR = 1.161 per year, 95% CI 1.096–1.231, *p* < 0.001), representing an approximately 16% increase in the odds of resistance per year (Figure 5). This is particularly concerning given the central role of amoxicillin/clavulanic acid as a first-line therapeutic agent in small animal practice.

Several classes showed borderline non-significant trends that did not survive Bonferroni correction, including Quinolones & Fluoroquinolones (OR = 0.944, *p*_adj = 0.525) and Cephalosporins 3rd/4th generation (OR = 0.937, *p*_adj = 0.295), suggesting a possible decreasing tendency that warrants monitoring in future surveillance studies (Figure 5).

No significant temporal trend was detected for overall MDR prevalence (OR = 0.966, 95% CI 0.913–1.022, *p*_adj = 1.000), indicating that despite shifts in resistance patterns within specific antibiotic classes, the overall proportion of multidrug-resistant isolates remained stable throughout the study period.

## 3. Discussion

The findings of this study underscore the critical importance of incorporating companion animals into antimicrobial resistance (AMR) surveillance programs, particularly within the framework of a One Health approach. Importantly, the present study highlights a concerning AMR profile of *Klebsiella* spp. isolated from dogs and cats, characterized by high resistance to commonly used antimicrobial classes, widespread multidrug resistance, and limited therapeutic options. In this study, *K. pneumoniae* generally exhibited higher resistance rates than *K. oxytoca*, particularly to amoxicillin/clavulanic acid, 1/2nd GC, 3/4th GC, fluoroquinolones, and tetracyclines. In both bacterial species, resistance rates were consistently higher among feline isolates than canine isolates. Overall, our findings are largely consistent with previous reports from other European countries, although some relevant differences in resistance levels and MDR prevalence were observed. In agreement with a previous retrospective study conducted in France, the low to moderate susceptibility to third- and fourth-generation cephalosporins observed in our study parallels the high resistance rates reported in France, supporting concerns regarding the empirical use of critically important β-lactams in veterinary medicine [15].

A notable contribution of the present study is the assessment of long-term antimicrobial resistance trends in *Klebsiella* spp. isolated from companion animals over a 10-year period. Overall, resistance patterns remained relatively stable throughout the study, although important changes were observed for specific antimicrobial classes. In *K. pneumoniae*, resistance to amoxicillin/clavulanic acid increased significantly over time, whereas resistance to aminoglycosides showed a significant decline. By contrast, resistance to aminopenicillins remained consistently high throughout the study period, reflecting the intrinsic resistance mechanisms of *Klebsiella* spp. and the limited therapeutic value of this antimicrobial class for *Klebsiella* infections. No significant temporal changes were detected for most other antimicrobial classes, including cephalosporins and fluoroquinolones, suggesting the persistence of resistant populations within companion animal settings.

The increase in resistance to amoxicillin/clavulanic acid is particularly concerning given the widespread use of this antimicrobial as a first-line treatment in small animal practice. Similar trends have been reported in European surveillance studies, where prolonged exposure to β-lactam/β-lactamase inhibitor combinations has been associated with the selection of strains carrying plasmid-mediated β-lactamases and other resistance determinants [21,22]. The increasing prevalence of ESBL-producing *K. pneumoniae* in companion animals may also contribute to the reduced effectiveness of this antimicrobial combination and highlights the need for routine susceptibility testing before treatment decisions.

Conversely, the decline in aminoglycoside resistance observed in *K. pneumoniae* may reflect changes in prescribing practices following the implementation of antimicrobial stewardship initiatives across Europe. Since the publication of the European Medicines Agency (EMA) recommendations on prudent antimicrobial use and the subsequent implementation of Regulation (EU) 2019/6, veterinary practitioners have been encouraged to restrict the use of critically important antimicrobials and adopt evidence-based prescribing strategies [23,24]. Similar reductions in resistance following decreased antimicrobial consumption have been documented in both human and veterinary settings [25]. A notable point of concordance among studies is the sustained efficacy of specific aminoglycosides. García-Fierro et al. reported very low resistance levels to amikacin and netilmicin (1.9–5.7%), despite high resistance rates to other aminoglycosides such as gentamicin and tobramycin [15]. Although resistance in our study was slightly higher overall, aminoglycosides remained among the antimicrobial classes with the highest in vitro susceptibility. This consistent finding across geographic regions supports their consideration as valuable therapeutic options, particularly when antimicrobial stewardship principles discourage the use of last-resort drugs such as polymyxins. However, these observations should be interpreted as surveillance data and should not replace individualized therapeutic decisions based on culture, susceptibility testing, and clinical evaluation.

The absence of significant temporal changes for fluoroquinolones and 3/4th GC is also noteworthy. These antimicrobials are classified as Category B (“Restrict”) by the EMA and should be reserved for situations where no suitable alternatives are available. Nevertheless, resistance levels remained moderate to high throughout the study period, indicating the persistence of resistant clones and resistance genes in companion animal populations. Similar observations have been reported in longitudinal studies of Enterobacterales from dogs and cats in Europe, where reductions in antimicrobial consumption were not always accompanied by rapid declines in resistance prevalence, likely due to the long-term maintenance of resistance determinants within bacterial populations and the environment [6].

Our results differ from the French study with respect to sulphonamides and fluoroquinolones [15]. While García-Fierro et al. reported resistance rates exceeding 80% for these classes, our data showed a slight declining trend over time, particularly for sulphonamides, although resistance to fluoroquinolones remained high (>30%). These discrepancies may reflect differences in antimicrobial usage patterns, study periods, or infection types across countries. Interestingly, Araújo et al. (2023) identified phenicols and carbapenems as the antimicrobial classes with the lowest resistance levels in companion animals in North of Portugal, which aligns with our findings regarding phenicols [26]. The high susceptibility observed for carbapenems further supports the need to preserve their efficacy. In the European Union, carbapenems are classified as antimicrobials reserved exclusively for the treatment of certain human infections and are prohibited for veterinary use, including companion animals, under Commission Implementing Regulation (EU) 2022/1255. Therefore, their continued activity should be regarded as an indicator of the limited dissemination of carbapenem resistance rather than as a therapeutic option in veterinary medicine [27].

The high prevalence of MDR isolates observed in our study (38% in dogs and 51% in cats) is comparable, though somewhat lower, than that reported in Portugal by Marques et al. [18], where 80% of *K. pneumoniae* isolates from urinary tract infections were MDR and *K. oxytoca* isolates were classified as XDR. The lower MDR prevalence in our dataset may be explained by the broader range of sample types included, whereas the Portuguese study focused specifically on UTIs, a clinical context often associated with intense antimicrobial exposure. More recent data from Portugal [26] showed MDR rates of 49% for *K. pneumoniae*, closely aligning with our findings, particularly in cats. The resistance profiles described in that study, including very high resistance to aminopenicillins, substantial resistance to first- and second-generation cephalosporins, and moderate to high resistance to third- and fourth-generation cephalosporins and fluoroquinolones, are largely consistent with our observations. However, their report of higher aminoglycoside resistance (41.7% to 66.7%) contrasts with the higher susceptibility observed in our study, suggesting regional variability or temporal changes in antimicrobial selection pressure. Finally, the predominance of MDR isolates in samples originating from wounds and the respiratory tract reported by Araújo et al. (2023) [26] is particularly relevant, as our study also identified reduced susceptibility to key antimicrobial classes in dermatological and respiratory samples, especially in cats, as previously observed Li et al. (2021) [20] in Spain [28]. This convergence highlights these infection sites as potential hotspots for resistant *Klebsiella* strains and underscores the importance of targeted surveillance and culture-based therapy.

Our findings are broadly consistent with previous European reports describing heterogeneous temporal changes in antimicrobial resistance among companion animal pathogens. A recent Italian study evaluating *Escherichia coli* and *Klebsiella pneumoniae* before and after the implementation of recent European antimicrobial stewardship measures also reported that resistance trends differed according to the antimicrobial class, supporting the concept that stewardship interventions may affect individual drug classes rather than overall multidrug resistance [29]. Likewise, the European multicentre study by Marques et al. demonstrated that temporal resistance patterns varied considerably between countries and antimicrobial classes, with decreases in fluoroquinolone resistance in some regions but increases in resistance to amoxicillin/clavulanate or gentamicin in others [30]. These observations agree with our results, in which aminoglycoside resistance declined significantly, whereas resistance to amoxicillin/clavulanic acid increased over time, while MDR prevalence remained stable. Together, these findings suggest that temporal changes in antimicrobial resistance are driven by antimicrobial-specific selective pressures and local prescribing practices rather than by uniform shifts across all antimicrobial classes.

This study has several limitations. As a laboratory-based retrospective analysis, it may be subject to referral bias, since samples are more likely to originate from animals with severe, recurrent, or treatment-failure infections. Geographic submission bias may also be present because sample distribution was not uniform across the study area. Furthermore, the dataset did not allow estimation of population-level prevalence, as only submitted clinical isolates were analyzed. The lack of detailed clinical information, including previous antimicrobial treatments and comorbidities, limited the evaluation of risk factors associated with resistance. Finally, resistance characterization was based solely on phenotypic testing, and molecular mechanisms underlying antimicrobial resistance were not investigated. Despite these limitations, the large number of isolates and extended study period provide valuable insights into antimicrobial resistance trends in *Klebsiella* spp. from companion animals.

In conclusion, the present findings corroborate European evidence indicating that *K. pneumoniae* in companion animals represents a significant and growing antimicrobial resistance challenge. The comparatively high susceptibility observed for aminoglycosides and phenicols suggests that resistance to these antimicrobial classes remains relatively uncommon among the isolates included in this study. Nevertheless, antimicrobial selection should always be based on bacterial culture and antimicrobial susceptibility testing, together with clinical judgment and antimicrobial stewardship principles. Whereas critically important category B antimicrobials, such as third-/fourth-generation cephalosporins and fluoroquinolones, exhibited low to moderate effectiveness, raising concerns about their empirical use and reinforcing the need for prudent antimicrobial use, routine susceptibility testing, and continued epidemiological monitoring in vet clinics. Lastly, though the present study does not assess transmission between animals and humans, since molecular epidemiological studies would be required to investigate such transmission pathways, these findings emphasize that continued surveillance of AMR in companion animals is essential to support One Health monitoring and stewardship initiatives.

## 4. Materials and Methods

### 4.1. Data Source and Management

A retrospective observational study was conducted using clinical microbiology data obtained from the Department of Veterinary Medicine of a private diagnostic laboratory in Barcelona, Spain (Laboratorio Echevarne S.L.). The dataset comprised 1386 *Klebsiella* isolates recovered between 2016 and 2024 from clinical samples submitted by veterinary hospitals and clinics located in Spain, Portugal, and Andorra.

Samples were eligible for inclusion when (i) bacterial identification was consistent with the genus *Klebsiella*, (ii) antimicrobial susceptibility testing had been performed, and (iii) information regarding animal species and sample origin was available. Records with incomplete microbiological or epidemiological information were excluded from the analysis.

Data cleaning was performed to identify and exclude incomplete records. To minimize potential bias associated with repeated sampling, duplicate isolates obtained from the same animal, collected during the same infection episode, and exhibiting an identical bacterial identification and antimicrobial susceptibility profile were excluded. When multiple isolates from the same animal were available, only the first isolate meeting the inclusion criteria was retained for analysis. Isolates recovered from different infection episodes or from different anatomical sites were considered independent observations and were therefore included. The following variables were extracted for analysis: animal species, sample origin, bacterial identification, antimicrobial susceptibility profile, and geographical location of each case. Sample origin was categorized as dermatological (dermatitis and skin infections), otic (otitis), respiratory (upper and lower respiratory tract infections), and others (including urinary, gastrointestinal, reproductive, bone, joint, cerebrospinal fluid, and ocular infections).

### 4.2. Bacterial Characterization & Antimicrobial Susceptibility Testing

Microbiological analysis and antimicrobial susceptibility testing were conducted by the laboratory following methodologies previously published by the authors [19,20,22]. Clinical samples submitted by veterinary clinics and hospitals were processed according to the routine diagnostic procedures of the laboratory. Samples were inoculated onto appropriate culture media according to the sample type and incubated under aerobic conditions at 35 ± 2 °C for 18–24 h. Colonies showing morphological characteristics compatible with Enterobacterales were subcultured to obtain pure isolates before identification.

Bacterial identification was performed using matrix-assisted laser desorption/ionization time-of-flight mass spectrometry (MALDI-TOF MS) (Bruker Daltonics, Bremen, Germany). Isolates identified as belonging to the genus *Klebsiella* were included in the study.

All microbiological procedures were performed within the quality management system of Laboratorio Echevarne, which has maintained ISO 9001 certification since 1998 [31] and is accredited by the Spanish National Accreditation Body (ENAC) according to the ISO/IEC 17025 standard [32] (Technical Annex 511/LE1947, Pharmaceutical Toxicology and Microbiology). Internal quality-control procedures and equipment calibration were conducted according to accreditation requirements.

Antimicrobial susceptibility testing (AST) was performed using the VITEK^®^ system following the Clinical and Laboratory Standards Institute (CLSI) guidelines. A total of 27 antimicrobial agents belonging to eight antimicrobial categories were evaluated. Inhibition zone diameters were interpreted according to the CLSI Performance Standards for Antimicrobial Susceptibility Testing of Bacteria Isolated from Animals (M31-A3/VET01, 2008). For antimicrobial agents lacking veterinary-specific interpretive criteria, the corresponding CLSI human clinical breakpoints (M100-S24, 2016) were applied, as previously described [19]. Quality-control testing was performed routinely using CLSI-recommended reference strains, including *Escherichia coli* ATCC 25922, to verify culture conditions and antimicrobial susceptibility testing performance. Results were accepted only when inhibition zone diameters for quality-control strains fell within the CLSI-established ranges.

The prevalence of multidrug resistance (MDR) was calculated for each species as the proportion of isolates classified as MDR according to the criteria of Magiorakos et al. (2012), implemented via the mdro() function of the AMR package (v2.1.1) in R studio with guideline = “CMI2012”, following the definitions set forth by EUCAST [33,34]. Isolates categorized as intermediate were considered non-susceptible for the purposes of resistance and multidrug-resistance analyses, in accordance with current epidemiological recommendations and the definitions proposed by Magiorakos et al. (2012) [34]. The prevalence of multidrug resistance (MDR) was calculated for each species as the proportion of isolates classified as MDR as previously described by Magiorakos et al. [34], implemented using the mdro() function of the AMR package (v2.1.1) in R with guideline = “CMI2012”. An isolate was considered resistant to an antimicrobial category when it was non-susceptible to at least one agent within that category. MDR isolates were defined as non-susceptible to at least one agent in three or more antimicrobial categories; extensively drug-resistant (XDR) isolates as non-susceptible to all but two or fewer categories; and pan-drug-resistant (PDR) isolates as non-susceptible to all tested antimicrobial categories.

### 4.3. Statistical Analyses

All statistical analyses were performed in RStudio (v4.4.1) [35]. Resistance rates were expressed as proportions with 95% confidence intervals calculated using the Wilson score method as implemented in the PropCIs package. Differences in resistance rates and MDR prevalence between species (dogs vs. cats) and between bacterial species (*K. pneumoniae* vs. *K. oxytoca*) were assessed using Fisher’s exact test. *p*-values were adjusted for multiple comparisons using the Bonferroni method.

The distribution of sample origins (dermatological, otic, respiratory, and other) between dogs and cats was compared using the Chi-square test, performed separately for each bacterial species and for all isolates combined.

Temporal trends in resistance were evaluated using two complementary approaches. First, logistic regression models were fitted for each antibiotic class and for MDR, with calendar year as a continuous independent variable and resistance status as the binary dependent variable. Odds ratios (ORs) per year with 95% confidence intervals were estimated using the logit link function. Second, the Cochran–Armitage trend test was applied to assess linear trends in resistance proportions across years. Both analyses were performed on all *Klebsiella* spp. isolates combined. *p*-values from both analyses were adjusted using the Bonferroni method. Temporal trends were additionally visualised, stratified by bacterial species for descriptive purposes; however, given the limited sample sizes per year when stratified, no formal statistical testing was performed at the animal species level for temporal analyses. A significance threshold of α = 0.05 was applied throughout.

## Figures and Tables

**Table 1:** Frequency and percentage (95% Confidence Interval) of *Klebsiella* spp. isolated from companion animals.

	Dog		Cat		Total	
Bacteria spp.	n	% (95% CI)	n	% (95% CI)	N	% (95% CI)
*Klebsiella pneumoniae*	458	68.4 (64.7–71.8)	105	75.5 (67.8–81.9)	563	69.6 (66.3–72.7)
*Klebsiella oxytoca*	135	20.1 (17.3–23.4)	25	18.0 (12.5–25.2)	160	19.8 (17.2–22.7)
*Klebsiella aerogenes*	38	5.7 (4.2–7.7)	5	3.6 (1.5–8.1)	43	5.3 (4.0–7.1)
Other *Klebsiella* spp. *	39	5.8 (4.3–7.9)	4	2.4 (1.1–7.2)	43	5.3 (4.0–7.1)
Total	670		139		809	

* *K. ornithinolytica*, *K. variicola*.

**Table 2:** EMA classification of antibiotic groups used for *K. pneumoniae* isolates in dogs and cats, ordered by treatment efficacy (S = sensibility) and grouped by sample origin.

DOGS (N = 410)	Susceptibility (%)	EMA	CATS (N = 92)	Susceptibility (%)	EMA
**Otic samples (n = 89)**			**Otic samples (n = 15)**		
Polymyxins	96.51	B	Phenicols	93.33	C
Aminoglycosides	90.5	C	Polymyxins	93.33	B
Sulfonamides	77.11	C	Aminoglycosides	84.62	C
Phenicols	77.01	C	Sulfonamides	69.23	C
Cephalosporins 3rd/4th	70.42	B	Fluoroquinolones	68.33	B
Fluoroquinolones	69.85	B	Cephalosporins 1st/2nd	60.98	C
Cephalosporins 1st/2nd	69.13	C	Tetracyclines	60	D
Tetracyclines	62.37	D	Amoxicillin/Clavulanic	53.33	C
Amoxicillin/Clavulanic	55.06	C	Cephalosporins 3rd/4th	52.17	B
Aminopenicillins *	0	D	Aminopenicillins *	0	D
**Dermatological samples (n = 185)**			**Dermatological samples ^£^ (n = 29)**		
Polymyxins	100	B	Aminoglycosides	75.58	C
Phenicols	84.42	C	Phenicols	68.18	B
Aminoglycosides	82.36	C	Tetracyclines	53.33	D
Sulfonamides	53.59	C	Cephalosporins 3rd/4th	49.38	B
Tetracyclines	50.67	D	Cephalosporins 1st/2nd	48.54	C
Fluoroquinolones	50.07	B	Sulfonamides	48.28	C
Cephalosporins 3rd/4th	46.96	B	Fluoroquinolones	46.85	B
Cephalosporins 1st/2nd	46.66	C	Amoxicillin/Clavulanic	27.59	C
Amoxicillin/Clavulanic	38.92	C	Aminopenicillins *	0	D
Aminopenicillins *	0	D			
**Respiratory samples (n = 90)**			**Respiratory samples ^£^ (n = 30)**		
Polymyxins	100	B	Aminoglycosides	75.28	C
Aminoglycosides	83.96	C	Phenicols	65.38	C
Phenicols	82.43	C	Sulfonamides	63.33	C
Sulfonamides	68.97	C	Tetracyclines	55.56	D
Tetracyclines	65.44	D	Fluoroquinolones	55.08	B
Fluoroquinolones	59.38	B	Cephalosporins 1st/2nd	52.43	C
Cephalosporins 3rd/4th	58.43	B	Cephalosporins 3rd/4th	51.81	B
Cephalosporins 1st/2nd	54.83	C	Amoxicillin/Clavulanic	43.33	C
Amoxicillin/Clavulanic	46.67	C	Aminopenicillins *	0	D
Aminopenicillins *	0	D			
**Others ^#^ (n = 46)**			**Others ^#^ (n = 18)**		
Polymyxins	100	B	Polymyxins	100	B
Phenicols	84.62	C	Aminoglycosides	79.59	C
Aminoglycosides	81.16	C	Phenicols	58.82	C
Sulfonamides	64.44	C	Sulfonamides	50	C
Tetracyclines	61.43	D	Cephalosporins 1st/2nd	40.68	C
Amoxicillin/Clavulanic	60.87	C	Amoxicillin/Clavulanic	38.89	C
Fluoroquinolones	58.89	B	Cephalosporins 3rd/4th	34.62	B
Cephalosporins 3rd/4th	54.62	B	Fluoroquinolones	28.57	B
Cephalosporins 1st/2nd	52.23	C	Tetracyclines	23.08	D
Aminopenicillins *	0	D	Aminopenicillins *	0	D

* Intrinsic resistance. **^£^** No polymyxins treatments recorded for dermatological and respiratory cases in cats. ^#^ Others category comprises minor cases such as urinary, gastrointestinal, reproductive, bone, joint, cerebrospinal fluid, and ocular infections.

**Table 3:** Frequency of MDR in *Klebsiella* isolates between dogs and cats.

Animal Species	MDR (n)	MDR (%)	95% CI	*p*-Value	*p*-Value Adjusted
Dog	183	38.4%	(34.1–42.8%)	0.0285 *	0.3425
Cat	48	51.1%	(41.1–60.9%)		

* Wilson score 95% confidence intervals were calculated for each proportion. Differences in MDR prevalence between dogs and cats were assessed using Fisher’s exact test.

**Table 4:** Logistic Regression Analysis of Temporal Trends in Antimicrobial Non-susceptibility of *Klebsiella* isolates from Dogs and Cats (2016–2024).

Antibiotic Class	OR per Year (95% CI)	*p*-Value	Adjusted *p*-Value
Aminopenicillins	1.266 (0.973–1.736)	0.092	1
Quinolones & Fluoroquinolones	0.944 (0.892–0.999)	0.048	0.525
Cephalosporins 1st/2nd	0.961 (0.907–1.018)	0.17	1
Cephalosporins 3rd/4th	0.937 (0.884–0.993)	0.027	0.295
Aminoglycosides	0.863 (0.809–0.921)	<0.001	<0.001 *
Amoxicillin/Clavulanic	1.161 (1.096–1.231)	<0.001	<0.001 *
Tetracyclines	1.011 (0.953–1.072)	0.727	1
Sulfonamides	1.013 (0.954–1.077)	0.667	1
Phenicols	1.043 (0.952–1.15)	0.38	1
Polymyxins	1.139 (0.769–2.145)	0.574	1
Multidrug Resistance (MDR)	0.966 (0.913–1.022)	0.227	1

OR: odds ratio per calendar year. CI: confidence interval; Bonferroni correction applied for 11 comparisons. Bonferroni correction for multiple comparisons. * Statistically significant (*p* < 0.05).

## Data Availability

The data presented in this study are available on request from the corresponding author. (The data are not publicly available due to privacy or ethical restrictions).
